# Interaction of Phenol-Soluble Modulins with Phosphatidylcholine Vesicles

**DOI:** 10.3390/pathogens1010003

**Published:** 2012-07-20

**Authors:** Anthony C. Duong, Gordon Y. C. Cheung, Michael Otto

**Affiliations:** Pathogen Molecular Genetics Section, Laboratory of Human Bacterial Pathogenesis, National Institute of Allergy and Infectious Diseases, The National Institutes of Health, 9000 Rockville Pike, Bethesda, MD 20892, USA; E-Mails: anthony.duong@nih.gov (A.C.D.); cheunggo@niaid.nih.gov (G.Y.C.C.)

**Keywords:** phenol-soluble modulin, *Staphylococcus aureus*, *Staphylococcus epidermidis*, toxin, vesicles

## Abstract

Several members of the staphylococcal phenol-soluble modulin (PSM) peptide family exhibit pronounced capacities to lyse eukaryotic cells, such as neutrophils, monocytes, and erythrocytes. This is commonly assumed to be due to the amphipathic, α-helical structure of PSMs, giving PSMs detergent-like characteristics and allowing for a relatively non-specific destruction of biological membranes. However, the capacities of PSMs to lyse synthetic phospholipid vesicles have not been investigated. Here, we analyzed lysis of synthetic phosphatidylcholine (1-palmitoyl-2-oleoyl-sn-glycero-3-phosphocholine, POPC) vesicles by all *Staphylococcus aureus* and *S. epidermidis* PSMs. In addition, we investigated the lytic capacities of culture filtrates obtained from different *S. aureus* PSM deletion mutants toward POPC vesicles. Our results show that all staphylococcal PSMs have phospholipid vesicle-lysing activity and the capacity of *S. aureus* culture filtrate to lyse POPC vesicles is exclusively dependent on PSMs. Notably, we observed largely differing capacities among PSM peptides to lyse POPC vesicles. Interestingly, POPC vesicle-lytic capacities did not correlate with those previously seen for the lysis of eukaryotic cells. For example, the β-type PSMs were strongly lytic for POPC vesicles, but are known to exhibit only very low lytic capacities toward neutrophils and erythrocytes. Thus our results also suggest that the interaction between PSMs and eukaryotic membranes is more specific than previously assumed, potentially depending on additional structural features of those membranes, such as phospholipid composition or yet unidentified docking molecules.

## 1. Introduction

Many members of the genus *Staphylococcus* are important human pathogens. *Staphylococcus aureus* in particular causes a multitude of frequently severe and life-threatening diseases, with acute disease promoted by a series of secreted toxins and other virulence determinants [1]. Coagulase-negative staphylococci, most notably *Staphylococcus epidermidis*, are a premier cause of hospital-associated infections on indwelling medical devices [2,3]. 

While virulence of *S. aureus* is clearly multi-factorial, the phenol-soluble modulin (PSM) peptide family has recently been identified as a key contributor to infection with highly virulent *S. aureus* strains, such as community-associated methicillin-resistant *S. aureus* (CA-MRSA) [4,5]. The PSMα peptides in particular strongly impact the capacity of CA-MRSA strains to lyse human neutrophils and other cell types, and promote skin infection and bacteremia. Less virulent hospital-associated strains characteristically produce smaller amounts of those peptide toxins [6].

In addition to their cytolytic potential, PSM peptides promote inflammatory responses by activating the FPR2 receptor [7]. Furthermore, they contribute to biofilm structuring, detachment, and the systemic dissemination of biofilm-associated infection [8,9]. Moreover, some PSMs may exhibit antimicrobial functions, for example toward *Streptococcus pyogenes* [10,11]. 

In contrast to the receptor-mediated pro-inflammatory roles of PSMs, the mechanistic basis of other PSM functions, such as cytolysis and biofilm maturation, is less well understood. Because cytolysis by PSMs is of utmost importance for staphylococcal virulence, the mechanism of PSM cytolytic activity is of key interest. Most likely, cytolysis is driven by the surfactant properties of PSMs, which are assumed to be critical for the formation of pores in cytoplasmic membranes and based on the characteristic amphipathic α-helical structure of PSMs. In fact, the PSM δ-toxin has been shown to lyse synthetic vesicles by the formation of short-lived pores [12]. However, other PSMs have never been analyzed for their potentials to lyse phospholipid vesicles. 

Phosphatidylcholine (POPC) is a major constituent of human cytoplasmic membranes. Membranes of red and white blood cells for example contain about equal amounts of cholesterol and phospholipids, among which POPC is the most abundant at 30% or higher of total membrane phospholipid [13,14]. Notably, POPC is present mostly in the outer leaflet of the human cytoplasmic membrane [15], and thus a predominant component of the membrane part interacting with exogenous toxins such as PSMs. In contrast, bacterial membranes do not contain cholesterol and have a phospholipid composition strongly different from that of eukaryotic membranes. *S. aureus* membranes for example contain mostly phosphatidylglycerol, diphosphatidylglycerol, and lysylphosphatidylglycerol, but no POPC [16]. 

Here, we analyzed the interaction of all PSM peptides known in *S. aureus* and *S. epidermidis*, and of *psm* isogenic deletion mutants of a CA-MRSA strain, with synthetic POPC vesicles. Our analyses demonstrate pore-forming activity of all PSM peptides. Importantly, PSM peptides without considerable reported cytolytic activities toward human cells, such as PSMβ peptides, lysed POPC vesicles very efficiently. This indicates that the interaction of PSMs with eukaryotic membranes is governed by more complex interactions than previously believed. The elucidation of such interactions will be an important task of future investigations aimed to answer the key question of why eukaryotic cells are much more susceptible to PSM cytolytic activity than bacterial cells.

## 2. Results and Discussion

To analyze whether and to which degree PSM peptides lyse POPC vesicles, we set up a fluorescein release assay. We tested final PSM peptide concentrations in the range of 0.25 to 2 μM and chose 0.5 and 1 μM for the final assays, as with those concentrations quantitative differences were most evident. The relative capacities of the peptides to lyse the vesicles were measured over time and curves were fit to a one-phase association. Association constants were determined to express differences in lytic capacities. 

### 2.1. Lytic Activities of PSM Peptides

**Figure 1:**
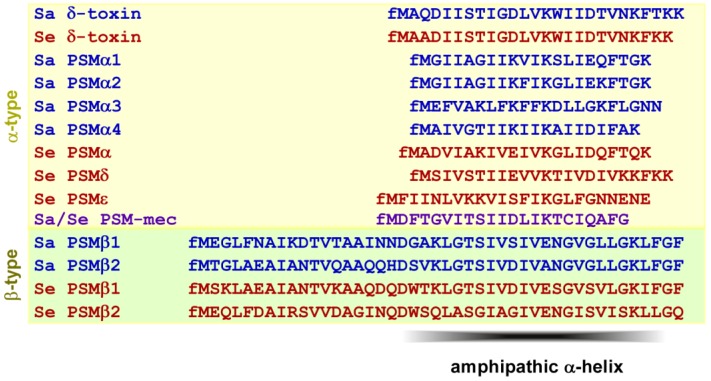
Amino acid sequences of PSM peptides of *S. aureus* and *S. epidermidis*. Note all PSM peptides carry an N-terminal N-formyl methionine (fM), as they are secreted without signal peptide. Sa, *S. aureus*; Se, *S. epidermidis*. PSM-mec is encoded on a methicillin resistance mobile genetic element present in some strains of *S. aureus* and *S. epidermidis*. PSMs of the α-type form an amphipathic α-helix. In the longer β-type PSMs, the C-terminal part contains an amphipathic α-helix.

**Figure 2:**
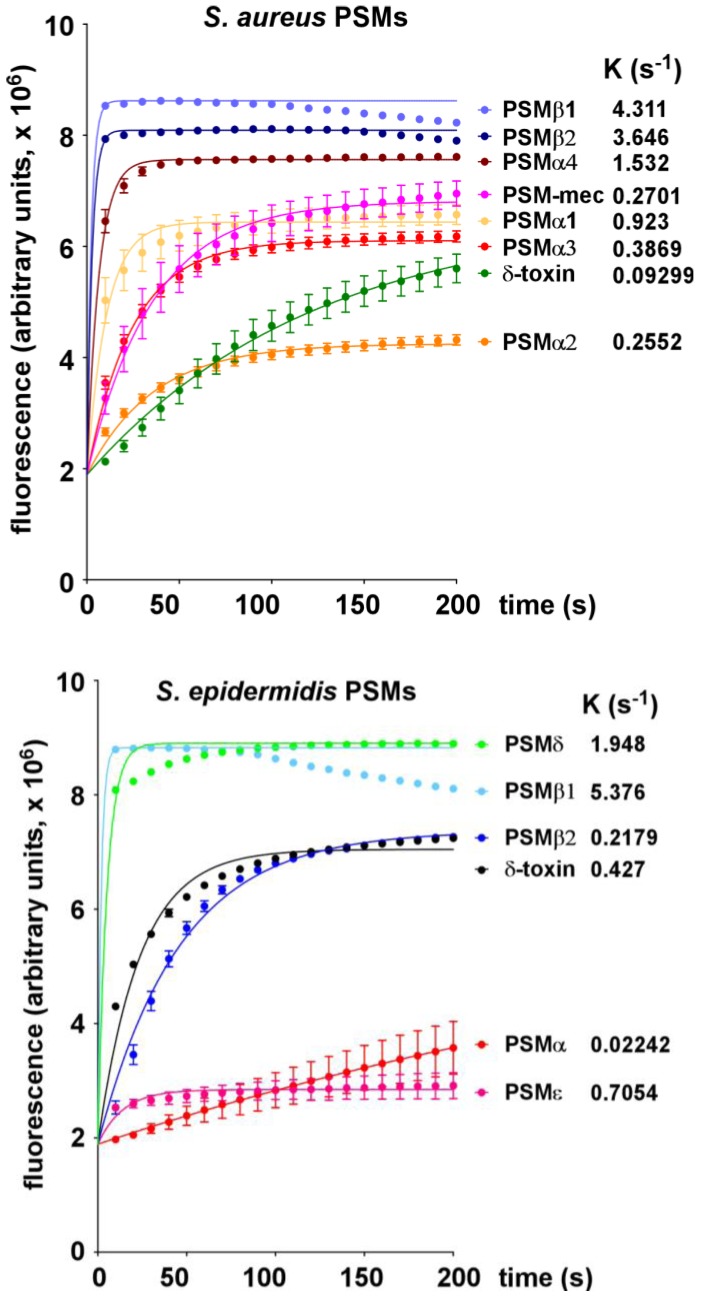
Lysis of POPC vesicles by *S. aureus* and *S. epidermidis* PSMs at 0.5 μM.

**Figure 3:**
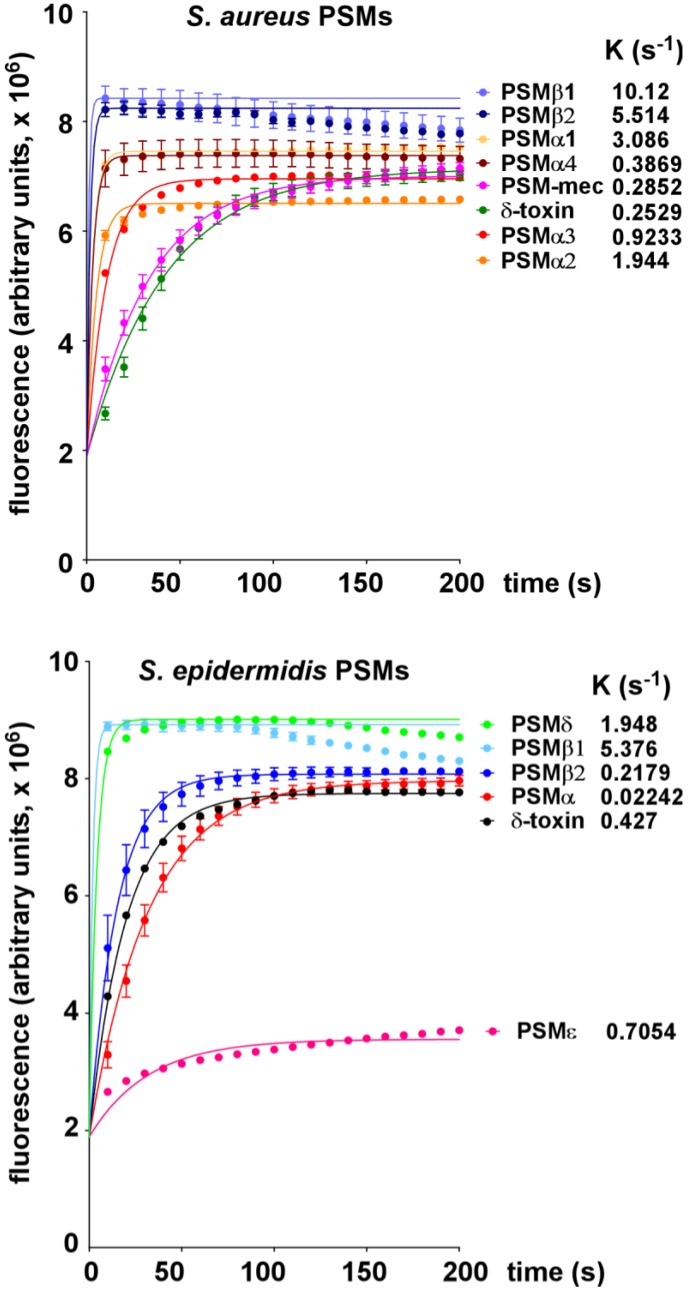
Lysis of POPC vesicles by *S. aureus* and *S. epidermidis* PSMs at 1 μM.

All PSM peptides of *S. aureus* and *S. epidermidis* (Figure 1) showed considerable potencies to lyse POPC vesicles. *S. aureus* PSM peptides lysed the POPC vesicles when applied at 0.5 μM final concentration in the order PSMβ1 > PSMβ2 > PSMα4 > PSMα1 > PSMα3 > PSMα2 > δ-toxin (Figure 2). At 1 μM final concentration, the order was similar, with only the different PSMα peptides changing order (PSMβ1 > PSMβ2 > PSMα1 > PSMα2 > PSMα3 > PSMα4 > δ-toxin) (Figure 2). The capacities of *S. epidermidis* PSM peptides to lyse the vesicles was in the order PSMβ1 > PSMδ > PSMε > δ-toxin > PSMβ2 > PSMα at both 0.5 and 1 μM (Figure 2 and Figure 3).

Notably, the relative capacities of *S. aureus* and *S. epidermidis* PSM peptides to lyse POPC vesicles are thus remarkably different from their capacities to lyse neutrophils and erythrocytes. Neutrophils are lysed by *S. aureus* PSMs in the order PSMα3 >> PSMα2, PSMα1, δ-toxin >> PSMα4, PSMβ1, PSMβ2 and *S. epidermidis* PSMs in the order PSMδ >> PSMε > δ-toxin > PSMα > PSMβ1, PSMβ2 [4,17]. Erythrocytes are lysed by *S. aureus* PSMs in the order PSMα3, PSMα2 > PSMα1, PSMα4, PSMβ1, PSMβ2, δ-toxin and *S. epidermidis* PSMs in the order PSMδ >> δ-toxin, PSMα, PSMε, PSMβ1 > PSMβ2 [4,17]. Thus, the strongest differences between lysis of eukaryotic cells and synthetic POPC vesicles were noted for the β-type peptides of *S. aureus* and *S. epidermidis*. Namely, while the β-type peptides were among the most potent to lyse POPC vesicles, they characteristically show almost no potential to lyse neutrophils and only a low potential to lyse erythrocytes [4,17]. Importantly, the latter is in accordance with the apparent lack of influence of PSMβ peptides on *S. aureus* skin infection and *S. aureus* and *S. epidermidis* bacteremia [4,9].

### 2.2. Lytic Activities of *S. aureus* Culture Filtrates

**Figure 4:**
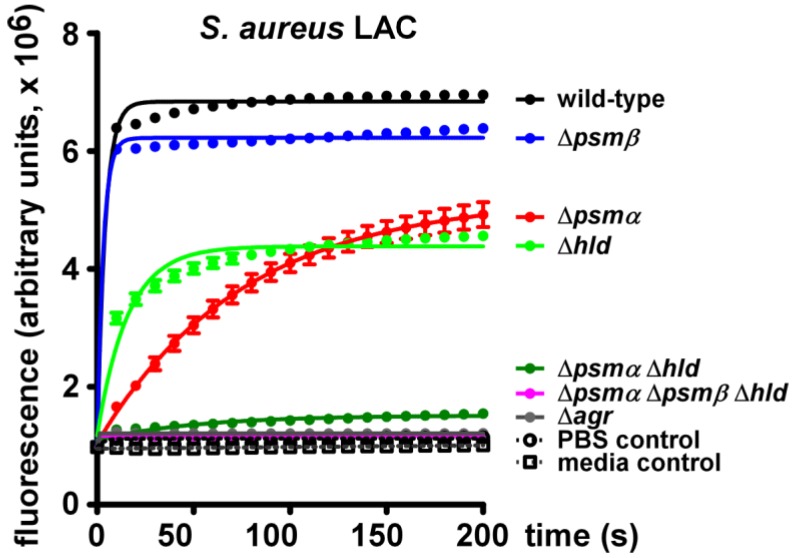
Lysis of POPC vesicles by culture filtrates of *S. aureus* LAC (USA300).

To analyze POPC-lysing activity of *S. aureus* and its dependence on the secretion of PSM peptides, we analyzed culture filtrates of the CA-MRSA strain LAC (USA300) and isogenic mutants in the *psm*α, *psm*β, or *hld* (δ-toxin) loci (Figure 4). We also used a mutant in which sequential deletion of all *psm* loci resulted in a completely PSM-free *S. aureus* [11]. Additionally, we analyzed an isogenic mutant in the *agr* system. Agr strictly regulates production of all PSMs and in *agr* mutants PSM concentrations are commonly below detection limits [4,18].

While culture filtrate of the USA300 wild-type strain showed strong capacity to lyse POPC vesicles, lysis was completely abolished when filtrates of the isogenic *agr* mutant were used. Lytic activity of the *agr* mutant strain culture filtrate was at the same low level as that observed with PBS or media controls. This finding demonstrates strong capacity of secreted molecules in *S. aureus* USA300 to lyse POPC vesicles and indicates that all lytic molecules are Agr-regulated. 

In addition to PSMs, Agr controls a large series of other secreted proteins, among them several toxins [19,20]. Thus, to determine whether the POPC-lytic activity of *S. aureus* USA300 is due to PSMs or other Agr-regulated proteins, we measured the lytic capacity of the PSM-free *psm*α/*psm*β/*hld* mutant, in which only PSMs but not other molecules are lacking. This mutant showed a very low level of released fluorescence that was indistinguishable of the levels observed with the *agr* mutant strain or the controls. Thus, these analyses demonstrated that the POPC-lytic activity of *S. aureus* USA300 is exclusively due to PSMs.

Next, we assayed *psm* mutant strains to determine which PSM peptides are primarily responsible for the lytic activity of *S. aureus* culture filtrate. The largest decrease in POPC-lytic activity was seen with culture filtrate of the *psm*α operon mutant (lacking PSMα1, PSMα2, PSMα3, and PSMα4). Culture filtrate of the δ-toxin mutant also had considerably decreased lytic activity. Contrastingly, lytic activity of the *psm*β operon mutant (lacking PSMβ1 and PSMβ2) was only slightly decreased compared to that of the wild-type. Accordingly, a mutant in the *psm*α and *hld* loci (only expressing PSMβ1 and PSMβ2) showed lytic activity that was only barely increased compared to the total *psm* deletion and *agr* culture filtrates, and to negative controls. At first glance, these results appear to contradict those obtained with synthetic PSM peptides (Figure 2 and Figure 3). However, one has to consider that PSMβ peptides are produced at much lower levels compared to PSMα peptides and δ-toxin [21]. Thus, POPC vesicle-lytic activity of *S. aureus* is primarily due to PSMα peptides and, to a somewhat lesser extent, δ-toxin.

## 3. Experimental Section

1-palmitoyl-2-oleoyl-sn-glycero-3-phosphocholine (POPC) vesicles were prepared as described previously [12]. Briefly, a POPC (Avanti Polar Lipids) lipid film, which was prepared after rotary evaporation and lyophilization, was rehydrated in a solution of 20 mM 3-(N-morpholino)propanesulfonic acid (Sigma), 100 mM potassium chloride (Sigma), 0.01 mM ethylene glycol tetraacetic acid (Sigma), 0.02% (w/v) sodium azide, 50 mM carboxyfluorescein (Fluka), and 100 mM potassium chloride (buffer 1). The hydrated lipids were passed through a polycarbonate and nitrocellulose membrane multiple times in a Mini-Extruder (Avanti Polar Lipids, Inc.). Lipid fractions were collected after the extruded material was added to a Sephadex G-25 column equilibrated with buffer 1 without carboxyfluorescein.

Lipid concentrations were measured using the Bartlett assay as described previously [12]. Purified lipid vesicles were diluted to 200 μM in equilibration buffer and used within a day of preparation.

Synthetic N-formylated *S. aureus* and *S. epidermidis* PSMs (GL Biochem Shanghai Ltd.) were constituted and diluted in dimethyl sulfoxide.

Culture filtrates, from clinical CA-MRSA isolate USA300 (clone LAC) and isogenic *psm* and *agr* deletion mutants, were collected from bacterial cultures in tryptone soy broth (TSB) inoculated 1:100 from pre-cultures grown overnight, and grown for 8 h at 37 °C, with shaking at 200 rpm. PSM production in the culture filtrates was analyzed by high-performance liquid chromatography and confirmed to be as described [11].

Carboxyfluorescein release from POPC lipid vesicles incubated with PSMs or culture filtrates was measured by fluorescence (excitation at 470_nm_, emission at 520_nm_) in a 96 well plate fluorimeter (VICTOR3, Perkin Elmer). 

Curve fitting was performed using Graph Pad Prism Version 5.04. All assays were performed in triplicate and error bars show ±SEM.

## 4. Conclusions

Our results demonstrate that all PSM peptides of *S. aureus* and *S. epidermidis* have the capacity to lyse synthetic phospholipid (POPC) vesicles. Of note, the POPC-lytic activity of *S. aureus* culture filtrate is exclusively dependent on PSMs. Furthermore, we noted considerable differences in the lytic capacities of different PSM peptides, which did not correlate with previously described capacities to lyse eukaryotic cells. These noticeable discrepancies, most pronounced for PSMβ peptides, indicate that the lytic activities that PSMs have toward human cells is not simply determined by surfactant-type interaction of PSMs with phospholipid bilayers. Elucidation of the molecular basis of these discrepancies, which may include differential composition of bacterial versus eukaryotic membranes or the requirement for specific docking structures, is an important task of future research. 
